# Cabotegravir for the prevention of HIV-1 in women: results from HPTN 084, a phase 3, randomised clinical trial

**DOI:** 10.1016/S0140-6736(22)00538-4

**Published:** 2022-05-07

**Authors:** Sinead Delany-Moretlwe, James P Hughes, Peter Bock, Samuel Gurrion Ouma, Portia Hunidzarira, Dishiki Kalonji, Noel Kayange, Joseph Makhema, Patricia Mandima, Carrie Mathew, Elizabeth Spooner, Juliet Mpendo, Pamela Mukwekwerere, Nyaradzo Mgodi, Patricia Nahirya Ntege, Gonasagrie Nair, Clemensia Nakabiito, Harriet Nuwagaba-Biribonwoha, Ravindre Panchia, Nishanta Singh, Bekezela Siziba, Jennifer Farrior, Scott Rose, Peter L Anderson, Susan H Eshleman, Mark A Marzinke, Craig W Hendrix, Stephanie Beigel-Orme, Sybil Hosek, Elizabeth Tolley, Nirupama Sista, Adeola Adeyeye, James F Rooney, Alex Rinehart, William R Spreen, Kimberly Smith, Brett Hanscom, Myron S Cohen, Mina C Hosseinipour, HPTN 084 study group, Aida Asmelash, Alice Sehurutshi, Allan Baguma, Anita Marais, Barbarah Kawoozo, Bongiwe Prudence Malinga, Brenda Gati Mirembe, Brenda Okech, Bryan Esterhuizen, Caroline Murombedzi, Daphne Gadama, Eldinah Hwengwere, Elizabeth Roos, Elizabeth S Magada, Emily Shava, Estelle Piwowar-Manning, Eunice Tahuringana, Felix GS Muhlanga, Francesca Conradie, Frank Angira, Gertrude Nanyonjo, Girisha Kistnasami, Hazzie Mvula, Ishana Naidoo, Jaco Horak, Jane Jere, Jeeva Moodley, Katie Shin, Kerry Nel, Kevin Bokoch, Lilian Birungi, Lynda Emel, Maletsatsi Monametsi, Marvelous Sibanda, Mercy Mutambanengwe, Miria Chitukuta, Moleen Matimbira, Muchaneta Bhondai-Mhuri, Ncamsile Sibisi, Neetha Morar, Netsai Mudzonga, Paul Natureeba, Paul Richardson, Petina Musara, Pippa Macdonald, Rejoice Nkambule, Repelang Mosime, Rhonda White, Ribka Berhanu, Ritha Ncube-Sihlongonyane, Rogers Sekabira, Samantha Siva, Saresha Pillay, Shamelle Govender, Sheiala Bamweyana, Siyabonga Nzimande, Steve Innes, Sufia Dadabhai, Taraz Samandari, Tchangani Tembo, Thandie Lungu Mabedi, Thandiwe Chirenda, Tinashe Chidemo, Victor Mudhune, Vikesh Naidoo, Wadzanai Samaneka, Yaw Agyei, Yeukai Musodza, Yolandie Fourie, Zakir Gaffoor

**Affiliations:** a Wits Reproductive Health and HIV Institute, University of the Witwatersrand, Johannesburg, South Africa; b Perinatal HIV Research Unit, University of the Witwatersrand, Johannesburg, South Africa; c Statistical Centre for HIV/AIDS Research and Prevention, Vaccine and Infectious Disease Division, Fred Hutchinson Cancer Research Center, Seattle, WA, USA; d Desmond Tutu TB Centre, University of Stellenbosch, Stellenbosch, South Africa; e Kisumu Clinical Research Site, Centre for Global Health Research, Kenya Medical Research Institute, Kisumu, Kenya; f Clinical Trials Research Centre, University of Zimbabwe, Harare, Zimbabwe; g HIV and other Infectious Diseases Research Unit, South African Medical Research Council, Durban, South Africa; h Blantyre Clinical Research Site, College of Medicine, University of Malawi, Blantyre, Malawi; i Botswana Harvard AIDS Institute Partnership (BHP), Gaborone, Botswana; j International AIDS Vaccine Initiative, Uganda Virus Research Institute, Entebbe, Uganda; k Baylor College of Medicine Children's Foundation Uganda, Kampala, Uganda; l Desmond Tutu Health Foundation, University of Cape Town, Cape Town, South Africa; m Makerere University–Johns Hopkins University Research Collaboration, Kampala, Uganda; n Eswatini Prevention Center, International Center for AIDS Care and Treatment Program at Columbia University Mailman School of Public Health, New York, NY, USA; o FHI 360, Durham, NC, USA; p Anschutz Medical Campus, University of Colorado, Aurora, CO, USA; q Department of Pathology, Johns Hopkins University School of Medicine, Baltimore, MD, USA; r Department of Medicine, Johns Hopkins University School of Medicine, Baltimore, MD, USA; s Department of Psychiatry, Stroger Hospital of Cook County, Chicago, IL, USA; t Division of AIDS, National Institute of Allergy and Infectious Diseases, Rockville, MD, USA; u Gilead Sciences, Foster City, CA, USA; v ViiV Healthcare, Durham, NC, USA; w University of North Carolina (UNC) at Chapel Hill, Chapel Hill, NC, USA; x UNC Project-Malawi, Lilongwe, Malawi

## Abstract

**Background:**

Oral pre-exposure prophylaxis has been introduced in more than 70 countries, including many in sub-Saharan Africa, but women experience considerable barriers to daily pill-taking, such as stigma, judgement, and the fear of violence. Safe and effective long-acting agents for HIV prevention are needed for women. We aimed to evaluate the safety and efficacy of injectable cabotegravir compared with daily oral tenofovir diphosphate plus emtricitabine (TDF-FTC) for HIV prevention in HIV-uninfected women.

**Methods:**

HPTN 084 was a phase 3, randomised, double-blind, double-dummy, active-controlled, superiority trial in 20 clinical research sites in seven countries in sub-Saharan Africa. Participants were eligible for enrolment if they were assigned female sex at birth, were aged 18–45 years, reported at least two episodes of vaginal intercourse in the previous 30 days, were at risk of HIV infection based on an HIV risk score, and agreed to use a long-acting reversible contraceptive method. Participants were randomly assigned (1:1) to either active cabotegravir with TDF-FTC placebo (cabotegravir group) or active TDF-FTC with cabotegravir placebo (TDF-FTC group). Study staff and participants were masked to study group allocation, with the exception of the site pharmacist who was responsible for study product preparation. Participants were prescribed 5 weeks of daily oral product followed by intramuscular injections every 8 weeks after an initial 4-week interval load, alongside daily oral pills. Participants who discontinued injections were offered open-label daily TDF-FTC for 48 weeks. The primary endpoints of the study were incident HIV infection in the intention-to-treat population, and clinical and laboratory events that were grade 2 or higher in all women who had received at least one dose of study product. This study is registered with ClinicalTrials.gov, NCT03164564.

**Findings:**

From Nov 27, 2017, to Nov 4, 2020, we enrolled 3224 participants (1614 in the cabotegravir group and 1610 in the TDF-FTC group). Median age was 25 years (IQR 22–30); 1755 (54·7%) of 3209 had two or more partners in the preceding month. 40 incident infections were observed over 3898 person-years (HIV incidence 1·0% [95% CI 0·73–1·40]); four in the cabotegravir group (HIV incidence 0·2 cases per 100 person-years [0·06–0·52]) and 36 in the TDF-FTC group (1·85 cases per 100 person-years [1·3–2·57]; hazard ratio 0·12 [0·05–0·31]; p<0·0001; risk difference –1·6% [–1·0% to –2·3%]. In a random subset of 405 TDF-FTC participants, 812 (42·1%) of 1929 plasma samples had tenofovir concentrations consistent with daily use. Injection coverage was 93% of the total number of person-years. Adverse event rates were similar across both groups, apart from injection site reactions, which were more frequent in the cabotegravir group than in the TDF-FTC group (577 [38·0%] of 1519 *vs* 162 [10·7%] of 1516]) but did not result in injection discontinuation. Confirmed pregnancy incidence was 1·3 per 100 person-years (0·9–1·7); no congenital birth anomalies were reported.

**Interpretation:**

Although both products for HIV prevention were generally safe, well tolerated, and effective, cabotegravir was superior to TDF-FTC in preventing HIV infection in women.

**Funding:**

National Institute of Allergy and Infectious Diseases, ViiV Healthcare, and the Bill & Melinda Gates Foundation. Additional support was provided through the National Institute of Mental Health, the National Institute on Drug Abuse, and the Eunice Kennedy Shriver National Institute of Child Health and Human Development. ViiV Healthcare and Gilead Sciences provided pharmaceutical support.

## Introduction

Women are disproportionately affected by HIV. In 2021, women and girls in sub-Saharan Africa accounted for three of five new HIV infections, and young women aged 15–24 years were three times as likely as young men in the same age group to be infected with HIV.^1^ In 2015, WHO recommended that tenofovir-based pre-exposure prophylaxis (PrEP) be offered as part of a comprehensive HIV prevention package to individuals at substantial risk of HIV infection.^2^ Since then, oral PrEP has been introduced in more than 70 countries, including many in sub-Saharan Africa. Despite expanding access to oral PrEP in the region, women experience considerable barriers to daily pill-taking, including stigma, judgement, and the fear of violence from partners, family, and community members.^3^, ^4^ Women face additional challenges compared with men who take PrEP consisting of tenofovir disoproxil fumarate plus emtricitabine (TDF-FTC); currently, only daily dosing is recommended in women to achieve optimal benefits for HIV prevention. Pharmacokinetic studies have shown lower concentrations of tenofovir diphosphate in vaginal tissues than in rectal tissues.^5^ Previous work has suggested that 6–7 doses of TDF-FTC per week are required to achieve vaginal tenofovir diphosphate concentrations associated with HIV protection.^5^

**Research in context**

**Evidence before this study**

New long-acting agents are needed to increase the options for HIV prevention. A published systematic review of eight studies of seven unique phase 2 randomised trials using long-acting injectable antiretroviral drugs found that cabotegravir, which was included in four of these studies, was generally safe and well tolerated, with adverse event and adverse event-related withdrawal rates similar to placebo. Pharmacokinetic–pharmacodynamic analyses of cabotegravir showed favourable viral inhibitory activity with a 600 mg intramuscular injection administered every 8 weeks.

**Added value of this study**

This large phase 3 trial provides the first evidence of the safety and efficacy of cabotegravir when used for HIV prevention in women. Cabotegravir was superior to daily oral tenofovir disoproxil fumarate plus emtricitabine in preventing HIV infection in this trial population.

**Implications of all the available evidence**

Antiretroviral pre-exposure prophylaxis is highly effective in preventing HIV infection in women at risk of HIV-1 infection. Cabotegravir is superior to oral tenofovir disoproxil fumarate plus emtricitabine and might overcome many of the challenges of daily oral PrEP in women.

Long-acting products such as cabotegravir, a novel integrase strand transfer inhibitor, might overcome many of these challenges when administered as an intramuscular injection every 8 weeks. Cabotegravir has potent antiretroviral activity and has been shown, in non-human primates, to prevent vaginal simian HIV infection.^6^, ^7^ Evidence from both treatment and early safety studies have confirmed that cabotegravir has an acceptable safety profile and supported further assessment of cabotegravir in phase 3 trials.^8^, ^9^ The HPTN 083 study showed that cabotegravir was safe and effective in preventing HIV in cisgender men and transgender women who have sex with men.^10^ The HPTN 084 trial was designed concurrently to HPTN 083 to evaluate the safety and efficacy of injectable cabotegravir compared with daily oral TDF-FTC for HIV prevention in HIV-uninfected women.

## Methods

### Study design and participants

HPTN 084 is a phase 3, randomised, double-blind, double-dummy, active-controlled, superiority trial done in 20 clinical research sites in seven countries in sub-Saharan Africa where the burden of HIV in women is high (ie, Botswana, Eswatini, Kenya, Malawi, South Africa, Uganda, and Zimbabwe). The protocol was reviewed and approved by national drug authorities and local research ethics committees in each of the 20 participating sites.

Participants were eligible for enrolment if they were assigned female sex at birth, were aged 18–45 years, reported at least two episodes of vaginal intercourse in the previous 30 days, were at risk of HIV infection based on an HIV risk score,^11^ and agreed to use a long-acting reversible contraceptive method with a failure rate of less than 1%. Participants were excluded if they were pregnant or breastfeeding; had substantial renal, hepatic, or cardiovascular disease; had a history of seizures, coagulopathy, or allergy to any of the study products; or if they were previously enrolled in an HIV vaccine or monoclonal antibody trial. All eligible participants were required to have non-reactive test results at the site with at least one HIV rapid antibody test cleared by the US Food and Drug Administration, a laboratory-based antigen–antibody test, and were required to have undetectable HIV RNA up to 14 days before enrolment. All participants provided written informed consent in a language of their choice before the start of any study procedures.

### Randomisation and masking

Randomisation was done using a permuted block of size 8, 10, or 12 (in random order) within each site. Study group assignment was electronically assigned at enrolment. Participants were randomly assigned (1:1) to either active cabotegravir with TDF-FTC placebo (cabotegravir group) or active TDF-FTC with cabotegravir placebo (TDF-FTC group). Each group received the assigned treatment plus a placebo representing the treatment that was not assigned (ie, double-dummy).

Placebo tablets were film-coated blue tablets that visually matched the TDF/FTC tablets. For injections, the placebo was similar looking to the active injection. For additional allocation concealment, a coloured overlay was wrapped around the syringe to maintain the masking.

Study staff and participants were masked to study group allocation, with the exception of the site pharmacist who was responsible for study product preparation.

### Procedures

**Figure 1:**
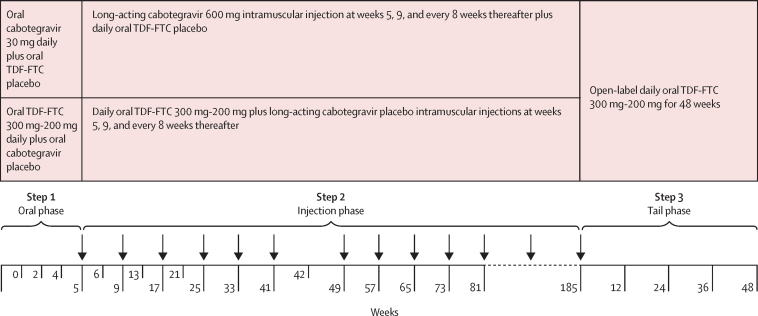
Trial design TDF-FTC=tenofovir disoproxil fumarate plus emtricitabine.

In step 1, participants initially received up to 5 weeks of daily oral product: either 30 mg cabotegravir and TDF-FTC placebo or co-formulated TDF-FTC 300 mg (TDF)-200 mg (FTC) and cabotegravir placebo (figure 1). This oral lead-in phase was included to confirm cabotegravir tolerability before injection. Participants returned at weeks 2 and 4 for safety assessments. Participants with reported adequate drug exposure (defined as at least 50% adherence by pill count) and with acceptable safety laboratory assessments were subsequently transitioned to the injection phase of the study (step 2). During this phase, participants received an initial single 3 mL intramuscular gluteal injection, a second injection 4 weeks later, and injections every 8 weeks thereafter for up to 185 weeks. Injections comprised either 600 mg (200 mg/mL) cabotegravir or placebo made of intralipid 20% fat emulsion. Participants were also supplied with oral pills (TDF-FTC or placebo according to study group) to be taken daily. Participants returned for safety assessments 1 week after the initial injection, and then 4 weeks after the second and third injections. Thereafter, safety assessments were done at each injection visit. An injection re-load (ie, two injections 4 weeks apart followed by injections every 8 weeks) was offered to participants who experienced injection delays of 8 weeks or more after their target visit date.

At each visit, participants were counselled and tested for HIV using one or two rapid antibody tests as well as a laboratory-based antigen–antibody immunoassay, and were also tested for pregnancy before product dispensation. Behavioural risk assessments were collected using a computer-assisted self-interview. Physical examination was performed, and adverse events were assessed. Specific injection site reaction assessments were done at weeks 6, 13, 21, and 42, although participants could report an injection site reaction at any visit. Blood and urine samples were collected for safety assessments and blood was stored. Testing for hepatitis C antibodies and fasting lipid profiles as well as urinalysis was performed annually. Adherence counselling was standardised and aligned with national PrEP programme guidelines. Participants received a comprehensive HIV prevention package, which included HIV risk-reduction counselling; offer of condoms, lubricants, and contraception; and treatment for symptomatic sexually transmitted infections. Non-immune participants were offered hepatitis B vaccination at week 6 and subsequently, according to manufacturer's guidelines. Every 6 months, sexually transmitted infection testing was done on cervico-vaginal samples for *Trichomonas vaginalis* (Osom Trichomonas Rapid Test, Sekisui Diagnostics, MA, USA), and *Chlamydia trachomatis* and *Neisseria gonorrhoea* using nucleic acid amplification testing; syphilis serology was also assessed and treatment provided for identified infections according to local guidelines.

Participants who discontinued injections were offered open-label daily TDF-FTC for 48 weeks as a precautionary measure to reduce the potential for HIV infection and emergent resistance in the context of declining cabotegravir concentrations. If a participant had a positive pregnancy test or a suspected pregnancy due to a lapse in contraception, injectable study product was withheld and daily open-label TDF-FTC offered while the pregnancy was confirmed on a second test 4 weeks later. If the pregnancy was confirmed, daily open-label TDF-FTC was continued through the duration of pregnancy and breastfeeding, and the participant was unmasked and continued quarterly follow-up visits through to pregnancy outcome. All pregnant participants were referred for a first trimester ultrasound for fetal anomaly assessment. Following pregnancy outcome or cessation of breastfeeding, the participant could return to her originally allocated study product, now unmasked. Live infants were assessed again 12 months after delivery for congenital anomalies.

If HIV seroconversion was suspected on the basis of a reactive HIV test at the site, the study product was withheld pending confirmation of HIV infection. Advice was sought from a committee masked to study product allocation who recommended further confirmatory HIV testing using one or more of the following tests: HIV RNA testing, HIV 1/2 discriminatory testing, and ultra-sensitive HIV DNA testing. If HIV infection was confirmed at the study site, additional samples were collected to assess CD4 cell count, viral load, and HIV drug resistance; participants were then followed up at quarterly visits for 1 year. Participants were linked to local HIV treatment and care programmes during this period. Retrospective testing was done at the HPTN Laboratory Center to confirm and characterise all HIV infections and more in-depth details are reported elsewhere.^12^ Incident HIV infections were categorised according to the time infection was detected relative to study product administration—ie, infections were categorised according to whether they occurred before enrolment, during the oral lead in, the injection phase, or in the absence of recent drug exposure. This categorisation system was developed for the HPTN 083 trial and applied to the HPTN 084 trial for comparison.^10^

In participants with HIV infection, plasma tenofovir and intraerythrocytic tenofovir diphosphate concentrations were quantified in the TDF-FTC group at the first HIV-positive visit and selected earlier visits; plasma cabotegravir concentrations were measured at all study visits for participants in the cabotegravir group. Drug concentrations were assessed at the HPTN Laboratory Center by liquid chromatography-tandem mass spectrometry; the lower limits of quantification were as follows: cabotegravir 0·025 μg/mL, tenofovir 0·31 ng/mL, and tenofovir diphosphate 31·3 fmol/punch.^12^ Plasma tenofovir and intraerythrocytic tenofovir diphosphate concentrations were also measured in a randomly selected cohort of 405 participants in the TDF-FTC group to evaluate oral TDF-FTC adherence. Participants were grouped into adherence categories on the basis of tenofovir and tenofovir diphosphate concentrations in accordance with established thresholds.^12^, ^13^, ^14^, ^15^, ^16^ For consistency with previous placebo-controlled trials of TDF-FTC, the Data Safety and Monitoring Board reviewed plasma tenofovir concentrations from the random adherence subset in the TDF-FTC group.

### Outcomes

The primary efficacy endpoint was incident HIV infection in steps 1 and 2 combined. Participants were counted as having had an incident event if infection was confirmed by the independent adjudication committee that was masked to study group assignment.^17^ This committee also established the date of the first HIV-positive visit, using all available site-based HIV test data and in accordance with a pre-specified endpoint definition. In accordance with the intention-to-treat (ITT) principle, infections were included in the group to which the participant was allocated, regardless of whether the study product was stopped or open-label TDF-FTC was being administered. The primary safety endpoint was a grade 2 or higher clinical or laboratory adverse event according to the Division of AIDS Table for Grading the Severity of Adult and Paediatric Adverse Events (version 2.1).^18^

Secondary endpoints included HIV incidence across all three steps of the trial (figure 1). HIV incidence was also assessed in pre-specified subgroups by age, contraceptive use method, body-mass index (BMI), and acceptability and willingness to use the study product. Tertiary outcomes included sexual risk behaviours, incident sexually transmitted infections, pregnancy incidence and outcomes, weight, and HIV drug resistance. This Article describes the primary outcomes and provides brief information for many of the secondary and tertiary study outcomes; additional information will be reported elsewhere.

### Statistical analysis

114 events were required to have 90% power to detect a hazard ratio (HR) of 0·54 for incident HIV infections in the cabotegravir group compared with the TDF-FTC group with a one-sided significance level of 0·025, assuming five pre-planned (four interim and one final) analyses. Assuming an incidence of 2·07 cases per 100 person-years in the TDF-FTC group, equal allocation to the groups, an average follow-up period of 2·6 years, and 5% loss to follow-up per year, a sample size of 3200 participants was considered robust against uncertainties in adherence rates.

The primary ITT analysis included all participants who were randomised and confirmed as HIV-uninfected at enrolment according to a site-based HIV testing algorithm. Incident infections that occurred during the oral (step 1) or injection phase (step 2) of the study were included in the primary analysis, regardless of product use. Cox proportional hazards modelling was used to estimate an HR and 95% CIs for incident HIV infection in the cabotegravir group compared with the TDF-FTC group, stratified by site and adjusted for the group-sequential design.^19^ Survival curves were generated on the basis of Kaplan-Meier estimates. Pre-specified subgroup analyses were done to assess the effects of age, contraceptive use, or BMI on the treatment effect using Firth's method, stratified by site. The primary safety analysis included all participants who had received at least one dose of study product. Missing data were considered missing completely at random.

In secondary and tertiary analyses, pregnancy and sexually transmitted infection incidence were estimated using Poisson regression with robust variance to model the number of incident outcomes per woman. A linear mixed model with time in study, treatment, and time by treatment interaction was fitted to evaluate mean weight gain by study group. We used SAS (version 9.4) for statistical analyses.

An independent Data Safety and Monitoring Board reviewed trial data every 6 months. Four interim analyses and a final analysis were planned; an O'Brien-Fleming spending function was used to determine stopping boundaries. At the planned second interim analysis on Nov 5, 2020, the Data Safety and Monitoring Board concluded that the pre-specified criteria for stopping of the trial due to efficacy had been met. The data presented in this Article reflect all data collected during the blinded phase of the trial up to and including Nov 5, 2020. Participants have since been unmasked and continued in follow-up on their original treatment assignment; participants are currently being offered the option to continue or initiate cabotegravir as part of a protocol amendment. This study is registered with ClinicalTrials.gov, NCT03164564.

### Role of the funding source

The funder of the study commented on the manuscript but had no role in study design, data collection, data analysis, and data interpretation.

## Results

**Figure 2:**
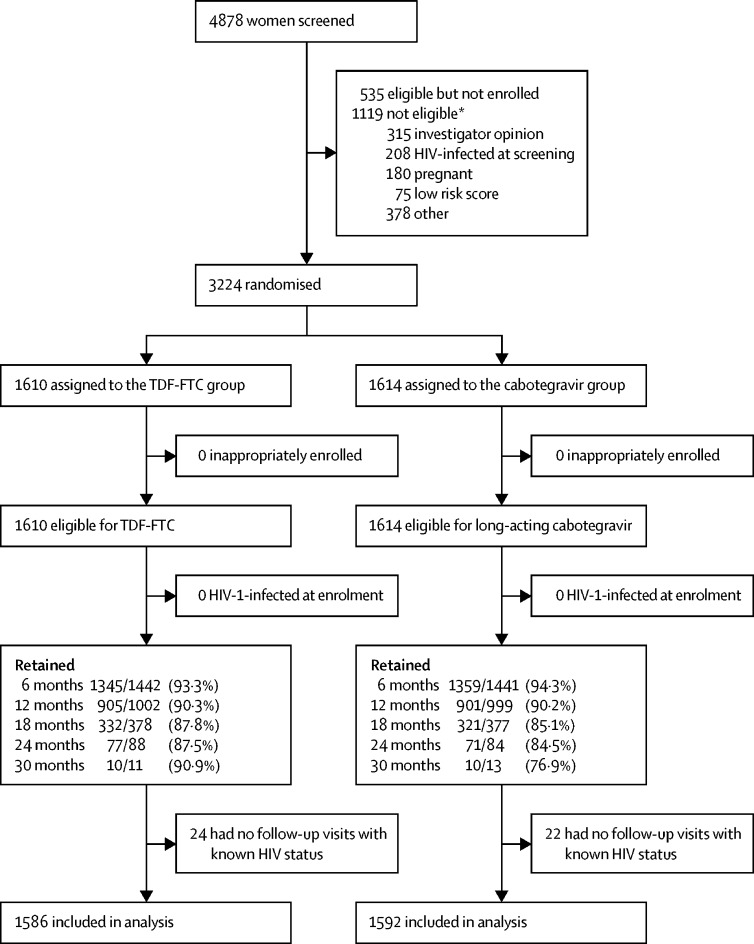
Trial profile TDF-FTC=tenofovir disoproxil fumarate plus emtricitabine. *One person could have had more than one reason for exclusion.

From Nov 27, 2017, to Nov 4, 2020, 4878 participants were assessed for eligibility; 1119 were excluded for ineligibility and 535 did not return for enrolment or declined to enrol (figure 2). The most common reasons for non-inclusion were a medical condition that in the opinion of the investigator might interfere with study participation (n=315), confirmed HIV infection (n=208), and current or intended pregnancy or breastfeeding during the study (n=180).

3224 participants were randomised (1614 in the cabotegravir group and 1610 in the TDF-FTC group). Of these, 46 participants had no follow-up visits with known HIV status. 3178 participants (1592 in the cabotegravir group and 1586 in the TDF-FTC group) who contributed 3898 person-years were included in the final efficacy analysis.

Overall, visit completion was high across both study groups despite disruption caused by the COVID-19 pandemic in 2020, with 1806 (90·3%) of 2001 planned visits completed at month 12 and 148 (86·0%) of 172 completed at month 24. The median follow-up time was 1·24 years (IQR 0·92–1·56) and did not vary by group. Enrolled participants received a median of eight injections (5–11) during this period. 195 (6·0%) of 3224 participants (85 [5·3%] of 1614 in the cabotegravir group and 110 [6·8%] of 1610 in the TDF-FTC group) discontinued the study product prematurely; discontinuation did not vary by study group.

Participants had a median age of 25 years (IQR 22–30; table 1). Most (3219 [99·8%] of 3224) self-identified as female. In the month before enrolment, 1755 (54·7%) of 3209 participants reported two or more sex partners, 1313 (40·9%) reported transactional sex, 1100 (34·3%) had a primary partner living with HIV or with an unknown HIV status, and 185 (5·8%) reported anal sex. The median HIV risk score at screening was 6 (5–7). 604 (18·9%) of 3189 had laboratory-diagnosed *C trachomatis,* 270 (8·6%) of 3133 had laboratory-diagnosed *T vaginalis*, and 210 (6·6%) of 3189 had laboratory-diagnosed *N gonorrhoeae* at enrolment; 103 (3·2%) of 3219 participants had reactive syphilis serology. Participant baseline characteristics were balanced by study group.

**Table 1:** Baseline characteristics of the intent-to-treat population

		**Cabotegravir group (n=1614)**	**TDF-FTC group (n=1610)**
Country			
	Botswana	46 (2·9%)	45 (2·8%)
	Eswatini	80 (5·0%)	80 (5%)
	Kenya	31 (1·9%)	35 (2·2%)
	Malawi	113 (7%)	111 (6·9%)
	South Africa	653 (40·5%)	655 (40·7%)
	Uganda	300 (18·6%)	296 (18·4%)
	Zimbabwe	391 (24·2%)	388 (24·1%)
Age, years		25 (22–30)	25 (22–20)
Aged <25 years		814 (50·4%)	816 (50·7%)
Race or ethnicity (self-reported)			
	Black African	1569 (97·2%)	1554 (96·5%)
	Asian	2 (0·1%)	3 (0·2%)
	Mixed race	2 (0·1%)	8 (0·5%)
	White	0	1 (0·1%)
	Other	41 (2·5%)	44 (2·7%)
Marital status			
	Married, civil union, or legal partnership	169 (10·5%)	174 (10·8%)
	Living with primary partner	106 (6·6%)	118 (7·3%)
	Not living with primary partner	869 (53·8%)	860 (53·4%)
	Single, divorced, or widowed	465 (28·8%)	454 (28·2%)
	Other	5 (0·3%)	4 (0·2%)
Education			
	No schooling	20 (1·2%)	12 (0·7%)
	Primary school	251 (15·6%)	255 (15·8%)
	Secondary school	1154 (71·5%)	1182 (73·4%)
	Technical training	48 (3·0%)	41 (2·5%)
	Tertiary education	141 (8·7%)	120 (7·5%)
Employed		451 (27·9%)	427 (26·5%)
Self-reported gender identity*			
	Female	1612 (99·9%)	1607 (99·8%)
	Male	0	3 (0·2%)
	Transgender male	2 (0·1%)	0
Sexual activity in past month†			
	≥2 sex partners	878/1609 (54·5%)	877/1600 (54·8%)
	Transactional sex	658/1609 (40·9%)	655/1600 (40·9%)
	Partner HIV-positive or unknown	542/1609 (33·7%)	558/1600 (34·9%)
	Anal sex	90/1609 (5·6%)	95/1600 (5·9%)
Modified VOICE risk score‡		6 (5–7)	6 (5–7)
Body-mass index ≥30 kg/m^2^		465 (28·8%)	430 (26·8%)
Sexually transmitted infections			
	*Chlamydia trachomatis*§	324/1602 (20·2%)	280/1587 (17·6%)
	*Neisseria gonorrhoeae*§	112/1602 (7·0%)	98/1587 (6·2%)
	*Trichomonas vaginalis*¶	141/1578 (8·9%)	129/1555 (8·3%)
	Positive syphilis serology‖	41/1611 (2·5%)	62/1608 (3·9%)

Data are mean (SD), n (%), or median (IQR). TDF-FTC=tenofovir disoproxil fumarate plus emtricitabine. VOICE=Vaginal and Oral Interventions to Control the Epidemic.^11^

* All participants were assigned female sex at birth.

† 15 missing (five in the cabotegravir group and ten in the TDF-FTC group) computer-assisted self-interview responses.

‡ Modified risk score excludes variables for curable sexually transmitted infections and HSV-2 serostatus.^11^

§ 35 results not done or invalid (12 in the cabotegravir group and 23 in the TDF-FTC group).

¶ 91 results invalid or not done (36 in the cabotegravir group and 55 in the TDF-FTC group).

‖ Five results missing or not done (three in the cabotegravir group and two in the TDF-FTC group); defined positive if both non-treponemal and treponemal test were reactive.

**Figure 3:**
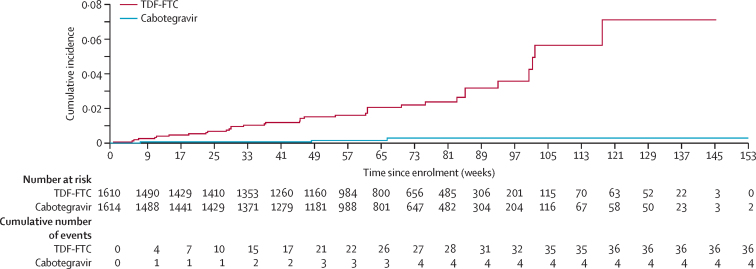
Cumulative HIV incidence by study group Kaplan-Meier estimates of HIV infection are shown. Four HIV infections were observed in the cabotegravir group (HIV incidence 0·20 per 100 person-years [95% CI 0·06–0·52]) and 36 in the TDF-FTC group (1·85 per 100 person-years [1·3–2·57]). Participants in the cabotegravir group had an 88% lower risk of HIV infection than those in the TDF-FTC group (hazard ratio 0·12 [0·05–0·31]; p<0·0001). TDF-FTC=tenofovir disoproxil fumarate plus emtricitabine.

**Figure 4:**
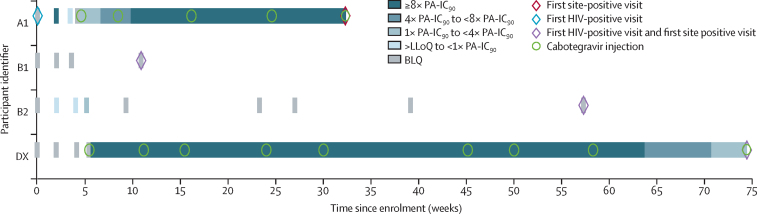
Pharmacological and virological data for HIV infections in the cabotegravir group The timing of key events for the four infections in the cabotegravir group are shown (participants A1, B1, B2, and DX). Group A includes infections that occurred before enrolment; group B includes infections that occurred with no recent exposure to cabotegravir; and group D includes infections that occurred in participants with appropriately timed cabotegravir injections. Participant DX acquired the infection during the step 2 injection phase but is called DX because three of nine injections were delayed. PA-IC90=protein-adjusted concentration required for 90% viral inhibition. LLoQ= lower limit of quantitation. BLQ=below the limit of quantitation.

40 HIV infections met the primary endpoint definition and were included in the ITT analysis. Four HIV infections were observed in the cabotegravir group (HIV incidence 0·20 per 100 person-years [95% CI 0·06–0·52]) and 36 in the TDF-FTC group (1·85 per 100 person-years [1·3–2·57]; table 2). Participants in the cabotegravir group had an 88% lower risk of HIV infection compared with those in the TDF-FTC group (HR 0·12 [0·05–0·31]; p<0·0001), after adjusting for site and the group-sequential design (figure 3). Analysis of treatment effect across the pre-specified subgroups of age, contraceptive use, and BMI at baseline were consistent with the primary outcome (table 2). The absolute risk difference between the cabotegravir and TDF-FTC groups was –1·6% (–1·0% to –2·3%). Two of four participants in the cabotegravir group with incident HIV infection had no evidence of recent cabotegravir exposure on history and did not receive any cabotegravir injections; cabotegravir concentrations were unquantifiable at the first HIV-positive visit in both people (participants B1 and B2; figure 4). In the third participant, HIV infection was detected at the study site during the injection phase and was initially classified as an incident infection; however, retrospective testing at the HPTN Laboratory Center indicated that this participant had HIV infection at study enrolment. This case was subsequently re-classified as a baseline infection (participant A1). The fourth infection occurred during the injection phase of the study in a participant with delayed injection visits (participant DX). This participant had cabotegravir concentrations of less than four times the protein-adjusted concentration required for 90% viral inhibition at the first HIV-positive visit; her last injection occurred 16·1 weeks before this visit.

**Table 2:** Cabotegravir effectiveness, overall and by subgroup

		**Events per total PY in cabotegravir group**	**Events per total PY in TDF-FTC group**	HR (95% CI)*	**p value**
Overall		4/1956 (0·20%)	36/1942 (1·85%)	0·12 (0·05–0·31)†	<0·0001
Age		..	..	..	0·53
	<25 years	3/866 (0·35%)	20/851 (2·34%)	0·17 (0·05–0·54)	..
	≥25 years	1/1090 (0·09%)	16/1091 (1·47%)	0·09 (0·02–0·49)	..
Contraceptive method		..	..	..	0·87
	DMPA	3/1009 (0·30%)	21/1000 (2·10%)	0·16 (0·05–0·53)	..
	NET-EN	1/175 (0·57%)	6/182 (3·30%)	0·22 (0·03–1·48)	..
	Implant	0	8/607 (1·32%)	0·06 (0·00–1·16)	..
	Other	0	1/152 (0·66%)	0·32 (0·01–9·89)	..
Body-mass index		..	..	..	..
	≤30 kg/m^2^	4/1389 (0·29%)	27/1447 (1·87%)	0·16 (0·06–0·45)	0·47
	>30 kg/m^2^	0	9/495 (1·82%)	0·05 (0·00–0·96)	..

HR=hazard ratio. PY=person-years. TDF-FTC=tenofovir disoproxil fumarate plus emtricitabine. DMPA=depot medroxyprogesterone acetate. NET-EN=norethisterone enanthate.

* Firth's method was used to estimate the HR and 95% CI when the subgroup had zero infections (used for subgroup analysis only, stratified by site).

† Median unbiased estimate HR (95% CI) for cabotegravir versus TDF-FTC overall displayed.

All 36 infections in the TDF-FTC group were incident infections; none were reclassified on post-hoc testing. None of these cases had tenofovir and tenofovir diphosphate concentrations consistent with 6–7 doses per week; only one participant had drug concentrations consistent with partial adherence (4–6 doses per week). Poor or non-adherence (<2 doses per week) was observed in most TDF-FTC incident infections (35 [98%] of 36). After exclusion of the baseline infection (participant A1), incidence in the cabotegravir group was recalculated, in a post-hoc analysis, as 0·15 per 100 person-years (95% CI 0·03–0·45). This resulted in a revised estimate of effect with even greater benefit observed in the cabotegravir group; HIV infection risk was reduced by 91% in the cabotegravir group compared with the TDF-FTC group (HR 0·09 [95% CI 0·04–0·27]; p<0·0001).

During the course of the trial, samples from a randomly selected cohort of 405 participants in the TDF-FTC group were evaluated for adherence to TDF-FTC. Overall, 1084 (55·9%) of 1939 evaluated samples yielded quantifiable plasma tenofovir concentrations (≥0·31 ng/mL), whereas 812 (41·9%) of 1939 had tenofovir concentrations consistent with daily use (≥40 ng/mL). However, intraerythrocytic tenofovir diphosphate concentrations revealed less consistent adherence over time, with unquantifiable concentrations observed in 456 (38·1%) of 1197 dried blood spot (DBS) samples tested; 215 (18%) of these 1197 samples had tenofovir diphosphate drug concentrations consistent with at least four doses per week over the past month (≥700 fmol/punch; appendix pp 1–2). By contrast, an estimated 3349 (93·1%) of 3599 person-years on study (1678 [93·0%] of 1805 in the cabotegravir group and 1671 [93·1%] of 1794 in the TDF-FTC group) were covered by injections—ie, cabotegravir or placebo injections were received on time or with a delay of less than 2 weeks.

No major integrase strand transfer inhibitor resistance mutations were detected in any of the four HIV infections observed in the cabotegravir group. One participant in the TDF-FTC group (E32) had a nucleoside reverse transcriptase inhibitor resistance mutation (M184V) detected. This participant had documented poor adherence to TDF-FTC before infection. Several participants in the TDF-FTC group also had non-nucleoside reverse transcriptase inhibitor resistance mutations detected (mainly K103N).

Overall, 2973 (92·2%) of 3224 participants who received at least one dose of study product reported a grade 2 or worse adverse event (table 3). There were no significant differences in the frequency of any grade 2 or higher adverse events by study group, apart from injection site reactions. Grade 3 or worse adverse events were also similar in frequency across both study groups (558 [17·3%] of 3224). Most of the grade 3 or higher adverse events were due to out-of-range laboratory values that were seldom clinically significant. For example, 7% of participants experienced a 30–50% decrease in creatinine clearance from baseline, but the majority maintained normal creatinine clearance (≥60 mL/min) and did not experience any study product interruptions. Overall, adverse events led to permanent discontinuation of blinded study medication in 40 (1·2%) participants (17 [1·1%] in the cabotegravir group and 23 [1·4%] in the TDF-FTC group) during steps 1 and 2. Adverse events of special interest including seizures (one [<0·1%] of 3224) or hepatic-related adverse events that led to product discontinuation (33 [1·0%]) were rare and did not differ by study group.

**Table 3:** Adverse events

		**Cabotegravir group (n=1614)**	**TDF-FTC group (n=1610)**
Grade 2 or higher adverse event		1487 (92·1%)	1486 (92·3%)
Most common adverse events (grade ≥2)*			
	Decreased creatinine clearance	1166 (72·2%)	1197 (74·3%)
	Gastrointestinal disorders†	334 (20·7%)	370 (23·0%)
	Increased creatinine	340 (21·1%)	330 (20·5%)
	Abnormal uterine bleeding†	311 (19·3%)	306 (19·0%)
	Headache†	276 (17·1%)	280 (17·4%)
	Upper respiratory tract infection†	276 (17·1%)	312 (19·4%)
	Chlamydia infection†	261 (16·2%)	287 (17·8%)
	Urinary tract infection†	229 (14·2%)	209 (13·0%)
	Increased amylase	169 (10·5%)	149 (9·3%)
	Decreased blood glucose	146 (9·0%)	146 (9·1%)
	Vulvovaginal candidiasis	127 (7·8%)	150 (9·3%)
	Gonococcal infection†	126 (7·8%)	125 (7·8%)
	Vulvovaginal trichomoniasis	124 (7·7%)	109 (6·8%)
	Back pain	106 (6·6%)	112 (7·0%)
	Blood creatinine phosphokinase	86 (5·3%)	69 (4·3%)
	Abnormal weight loss	85 (5·3%)	107 (6·6%)
	Vaginal discharge	83 (5·1%)	58 (3·6%)
	Nasopharyngitis	75 (4·6%)	86 (5·3%)
	Decreased blood phosphorous	58 (3·6%)	83 (5·2%)
Grade 3 or higher adverse event		276 (17·1%)	280 (17·4%)
Most common adverse events (grade ≥3)‡			
	Decreased creatinine clearance	110 (6·8%)	125 (7·8%)
	Increased creatinine	73 (4·5%)	67 (4·2%)
	Increased creatine phosphokinase	41 (2·5%)	32 (2·0%)
	Abnormal weight loss	21 (1·3%)	36 (2·2%)
	Increased alanine aminotransferase	11 (0·7%)	15 (0·9%)
	Increased aspartate aminotransferase	14 (0·9%)	13 (0·8%)
Adverse events of special interest			
	Seizure	0	1 (0·1%)
	Hepatic-related discontinuation	15 (0·9%)	18 (1·1%)
Serious adverse events		33 (2·0%)	33 (2·0%)
Deaths		3 (0·2%)	0
Injection site reactions§			
	Any	577/1519 (38·0%)	163/1516 (10·8%)
	Grade ≥2	192/1519 (12·6%)	25/1516 (1·6%)

Table cut based on imputed date of onset date when date is missing without missing month and year. Includes adverse events assigned MedRA codes by clinic staff. For participants reporting multiple events of the same MedRA term, the highest grade is counted. TDF-FTC=tenofovir disoproxil fumarate plus emtricitabine.

* Includes only adverse events reported by at least 5% of participants in either study group.

† Contains grouping of similar MedRA terms that fall within this preferred term.

‡ Includes only adverse events reported by at least 1% of participants in either study group.

§ Includes only participants who received one or more injections (ie, 1519 in the cabotegravir group and 1516 in the TDF-FTC group).

66 participants experienced at least one serious adverse event; 33 participants in the cabotegravir group and 33 in the TDF-FTC group. Only six serious adverse events were considered by the investigators to be related to study product (two in the cabotegravir group and four in the TDF-FTC group). The two serious adverse events in the cabotegravir group included hospitalisation for fetal distress and respiratory tract infection. The four serious adverse events in the TDF-FTC group included hospital admissions for investigation of hepatotoxicity (n=1) or raised transaminases (n=2), and seizure (n=1). There were three deaths in the study, all in the cabotegravir group. None of the three deaths observed in the study were attributed to study product; these deaths were due to hypertensive heart disease (n=1), a cerebrovascular accident (n=1), and an unexplained headache that could not be further investigated (n=1).

Injection site reactions were reported in 577 (38·0%) of 1519 participants in the cabotegravir group compared with 163 (10·8%) of 1516 in the TDF-FTC group (table 3). Of these, grade 2 or worse adverse events were experienced by 192 (12·6%) of 1519 in the cabotegravir group compared with 25 (1·6%) of 1516 in the TDF-FTC group. Pain was the most commonly reported symptom, with 570 (4·4%) of 12 901 injections in the cabotegravir group associated with pain compared with 146 (1·1%) of 12825 injections in the TDF-FTC group. Most injection site reactions were reported at the first injection and diminished over time. In the cabotegravir group, injection site reactions were reported in 438 (28·8%) of 1519 participants at the first injection; this decreased to 25 (1·9%) of 1322 participants by the fourth injection. There were no discontinuations of study product due to injection site reactions.

Overall confirmed pregnancy incidence in the trial was low (1·3 per 100 person-years [95% CI 0·9–1·7]) and did not appear to differ meaningfully by study group. There were 49 confirmed pregnancies: 29 in the cabotegravir group (1·5 per 100 person-years [1·0–2·2]) and 20 in the TDF-FTC group (1·0 per 100 person-years [0·6–1·6]). Outcome data were available for 31 (63%) of 49 pregnancies at the time of data lock, with the remainder of pregnancies ongoing. Most pregnancies resulted in a livebirth (13 of 18 in the cabotegravir group and 10 of 13 in the TDF-FTC group), with the remainder ending in pregnancy loss (spontaneous or induced). No congenital anomalies were observed.

The incidence of chlamydia was 19·6 per 100 person-years (95% CI 18–21). The incidence of gonorrhoea was 7·7 per 100 person-years (6·8–8·7); incidence did not vary significantly by study group.

There was evidence of an increase in weight in both study groups. In the cabotegravir group, there was a small but significant increase in average initial weight gain relative to the TDF-FTC group (0·4 kg [95% CI 0·27–0·51]; p<0·0001; appendix pp 1–3). Subsequently both groups showed weight gain, with a mean increase of 2·4 kg per year (1·9–3·0) in the cabotegravir group compared with 2·1 kg per year (1·9–2·4) in the TDF-FTC group (p=0·041).

## Discussion

The HPTN 084 trial showed that cabotegravir administered intramuscularly every 8 weeks was effective in preventing HIV infection in women at substantial risk for acquiring HIV in sub-Saharan Africa. These findings are consistent with results from the HPTN 083 trial, which was conducted in cisgender men and transgender women who have sex with men.^10^ HIV infection risk was 88% lower in the cabotegravir group, showing the superiority of cabotegravir over TDF-FTC in preventing HIV in this population. The pooled HIV incidence of 1 per 100 person-years in HPTN 084 is in stark contrast to HIV incidence rates of 3–4 per 100 person-years observed in other recent HIV prevention trials conducted in sub-Saharan Africa and supports the effectiveness of both study agents for PrEP in women.^20^, ^21^, ^22^

Cabotegravir provided an adherence advantage over daily oral pill-taking. An injection every 8 weeks is convenient and discreet, and might overcome the barriers to daily oral pill-taking, such as habit formation, difficulties with pill swallowing, HIV stigma, intimate partner violence, and discrimination.^4^, ^23^, ^24^ This is supported by our observation of less consistent dosing based on tenofovir diphosphate concentrations in DBS, which reflect cumulative drug exposure in the previous 6–8 weeks.^13^, ^14^ Most participants in the TDF-FTC group who acquired HIV infection had unquantifiable tenofovir and tenofovir diphosphate concentrations at the time of HIV infection. Studies show that many women in sub-Saharan Africa would prefer a long-acting injectable agent for PrEP.^25^, ^26^, ^27^ This might in part reflect the widespread use of and familiarity with injectable contraception. The protective benefit of cabotegravir was consistent across age groups, including in those younger than 25 years. Adolescent girls and young women in sub-Saharan Africa are at particularly high risk for HIV infection but in placebo-controlled trials of user-dependent PrEP agents, such as oral pills and vaginal rings, they have not derived benefits for HIV protection.^28^, ^29^ Cabotegravir might be an important new PrEP option for this population in particular. The current study was limited to women aged 18 years and older; studies to assess the safety and acceptability of cabotegravir in adolescents younger than 18 years and who weigh at least 35 kg are currently under evaluation (NCT04692077 and NCT04824131).

Cabotegravir was generally safe and well tolerated in this population, with no difference in the frequency of adverse events when compared with the TDF-FTC group, apart from injection site reactions. Although one in three participants in the cabotegravir group reported an injection site reaction, none of the participants discontinued injections for this reason. Concerns about integrase strand transfer inhibitor-associated weight gain have been reported and were of particular concern in this healthy, HIV-negative population, in which a quarter of participants were already classified as obese (BMI ≥30 kg/m^2^) at baseline.^30^ We observed a mean weight gain of about 2 kg per year across both groups of the trial, with no meaningful difference by trial group. These results are consistent with the findings from the HPTN 077 and HPTN 083 trials, which also did not observe significant weight gain overall that could be ascribed to cabotegravir; in HPTN 077, weight gain in the cabotegravir group was the same as placebo.^10^, ^31^ Despite limiting enrolment to women using long-acting reversible contraception, we confirmed 49 pregnancies (29 of which occurred in the cabotegravir group). To our knowledge, none of these pregnancies were associated with neural tube defects or other congenital anomalies. We are continuing to study the safety and pharmacokinetics of injectable cabotegravir during pregnancy and breastfeeding in the unblinded phase of the study. Both the unblinded phase and the open-label extension will provide additional opportunities for safety data collection beyond the median 1·2 years of follow-up in the blinded trial. We acknowledge that early stopping of the blinded portion of the trial limited the ability to assess blinded safety outcomes over 24 months.

Very few HIV infections were observed in the cabotegravir group during the injection phase of the study. In one of four cases, retrospective post-hoc testing revealed that the participant had HIV infection at enrolment. Detection of HIV infection at study sites was delayed in several participants in both this trial and in HPTN 083.^12^, ^17^ These instances illustrate the challenge of using conventional testing approaches to screen for HIV infection in studies using potent, long-acting, PrEP agents. More data are needed to establish the optimal approach for detection of HIV infection in people using cabotegravir and other long-acting agents for PrEP. In HPTN 083, five participants receiving cabotegravir had integrase strand transfer inhibitor resistance mutations detected with an ultrasensitive clinical assay that were ascribed to cabotegravir exposure; no integrase strand transfer inhibitor resistance mutations were detected in HPTN 084.^10^, ^12^, ^32^ Viral load testing might be a substantial barrier to implementation of cabotegravir in many settings in low-income and middle-income countries. More data are needed to understand potential differences in risk of breakthrough infection in cisgender women compared with cisgender men and transgender women who have sex with men, as well as subsequent resistance and response to dolutegravir-based treatment regimens that are widely used in sub-Saharan Africa.

In summary, the HPTN 084 trial provides robust evidence that cabotegravir has an acceptable safety profile, is well tolerated, and is superior to TDF-FTC in preventing HIV infection in women in sub-Saharan Africa. Given the urgent need for an expanded range of effective options for HIV prevention in women, these data support the inclusion of injectable cabotegravir as an additional choice, particularly for women in high-incidence settings where the need is greatest.

## Data sharing

Data collected for this study may be made available on request. The data archive will be held at the Fred Hutch Cancer Center (Seattle, WA, USA). Requests can be sent to HPTN-Data-Access@scharp.org.

**This online publication has been corrected. The corrected version first appeared at thelancet.com on May 5, 2022**

## Declaration of interests

PLA has received fees from Merck, ViiV Healthcare, and Gilead Sciences, and research support from Gilead Sciences paid to his institution. AR, WRS, and KS are employees of ViiV Healthcare. JFR is an employee of Gilead Sciences. All other authors declare no competing interests.

## References

[1] UNAIDS (July 14, 2021). Confronting inequalities: lessons for pandemic responses from 40 years of AIDS. https://www.unaids.org/en/resources/documents/2021/2021-global-aids-update-slideset.

[2] WHO (2015).

[3] Celum CL, Delany-Moretlwe S, Baeten JM (2019). HIV pre-exposure prophylaxis for adolescent girls and young women in Africa: from efficacy trials to delivery. J Int AIDS Soc.

[4] Velloza J, Khoza N, Scorgie F (2020). The influence of HIV-related stigma on PrEP disclosure and adherence among adolescent girls and young women in HPTN 082: a qualitative study. J Int AIDS Soc.

[5] Cottrell ML, Yang KH, Prince HM (2016). A translational pharmacology approach to predicting outcomes of preexposure prophylaxis against HIV in men and women using tenofovir disoproxil fumarate with or without emtricitabine. J Infect Dis.

[6] Andrews CD, Yueh YL, Spreen WR (2015). A long-acting integrase inhibitor protects female macaques from repeated high-dose intravaginal SHIV challenge. Sci Transl Med.

[7] Radzio J, Spreen W, Yueh YL (2015). The long-acting integrase inhibitor GSK744 protects macaques from repeated intravaginal SHIV challenge. Sci Transl Med.

[8] Markowitz M, Frank I, Grant RM (2017). Safety and tolerability of long-acting cabotegravir injections in HIV-uninfected men (ECLAIR): a multicentre, double-blind, randomised, placebo-controlled, phase 2a trial. Lancet HIV.

[9] Landovitz RJ, Li S, Grinsztejn B (2018). Safety, tolerability, and pharmacokinetics of long-acting injectable cabotegravir in low-risk HIV-uninfected individuals: HPTN 077, a phase 2a randomized controlled trial. PLoS Med.

[10] Landovitz RJ, Donnell D, Clement ME (2021). Cabotegravir for HIV prevention in cisgender men and transgender women. N Engl J Med.

[11] Balkus JE, Brown E, Palanee T (2016). An empiric HIV risk scoring tool to predict HIV-1 acquisition in African women. J Acquir Immune Defic Syndr.

[12] Eshleman SH, Fogel JM, Piwowar-Manning E, et al. Characterization of HIV infections in women who received injectable cabotegravir or tenofovir disoproxil fumarate/emtricitabine for HIV prevention: HPTN 084. *J Infect Dis* (in press). 10.1093/infdis/jiab576 PMC9113509 35301540

[13] Castillo-Mancilla JR, Zheng JH, Rower JE (2013). Tenofovir, emtricitabine, and tenofovir diphosphate in dried blood spots for determining recent and cumulative drug exposure. AIDS Res Hum Retroviruses.

[14] Anderson PL, Liu AY, Castillo-Mancilla JR (2017). Intracellular tenofovir-diphosphate and emtricitabine-triphosphate in dried blood spots following directly observed therapy. Antimicrob Agents Chemother.

[15] Hendrix CW, Andrade A, Bumpus NN (2016). Dose frequency ranging pharmacokinetic study of tenofovir-emtricitabine after directly observed dosing in healthy volunteers to establish adherence benchmarks (HPTN 066). AIDS Res Hum Retroviruses.

[16] Donnell D, Baeten JM, Bumpus NN (2014). HIV protective efficacy and correlates of tenofovir blood concentrations in a clinical trial of PrEP for HIV prevention. J Acquir Immune Defic Syndr.

[17] Marzinke MA, Grinsztejn B, Fogel JM (2021). Characterization of human immunodeficiency virus (HIV) infection in cisgender men and transgender women who have sex with men receiving injectable cabotegravir for HIV prevention: HPTN 083. J Infect Dis.

[18] US Department of Health and Human Services (July, 2017). Division of AIDS (DAIDS) table for grading the severity of adult and pediatric adverse events, corrected version 2.1. https://rsc.niaid.nih.gov/sites/default/files/daidsgradingcorrectedv21.pdf.

[19] Emerson SS, Fleming TR (1990). Parameter estimation following group sequential hypothesis testing. Biometrika.

[20] Corey L, Gilbert PB, Juraska M (2021). Two randomized trials of neutralizing antibodies to prevent HIV-1 acquisition. N Engl J Med.

[21] Gray GE, Bekker LG, Laher F (2021). Vaccine efficacy of ALVAC-HIV and bivalent subtype C gp120-MF59 in adults. N Engl J Med.

[22] Palanee-Phillips T, Baeten J, Heller K, et al. HIV incidence remains high in women in South Africa: data from the ECHO trial. 10th International AIDS Society Conference on HIV Science; July 21–24, 2019 (abstr LBPEC23).

[23] Pillay D, Stankevitz K, Lanham M (2020). Factors influencing uptake, continuation, and discontinuation of oral PrEP among clients at sex worker and MSM facilities in South Africa. PLoS One.

[24] Bärnighausen K, Geldsetzer P, Matse S (2021). Qualitative accounts of PrEP discontinuation from the general population in Eswatini. Cult Health Sex.

[25] Quaife M, Eakle R, Cabrera Escobar MA (2018). Divergent preferences for HIV prevention: a discrete choice experiment for multipurpose HIV prevention products in South Africa. Med Decis Making.

[26] Minnis AM, Browne EN, Boeri M (2019). Young women's stated preferences for biomedical HIV prevention: results of a discrete choice experiment in Kenya and South Africa. J Acquir Immune Defic Syndr.

[27] Laher F, Salami T, Hornschuh S (2020). Willingness to use HIV prevention methods among vaccine efficacy trial participants in Soweto, South Africa: discretion is important. BMC Public Health.

[28] Marrazzo JM, Ramjee G, Richardson BA (2015). Tenofovir-based preexposure prophylaxis for HIV infection among African women. N Engl J Med.

[29] Baeten JM, Brown ER, Hillier SL (2017). Dapivirine vaginal ring for HIV-1 prevention. N Engl J Med.

[30] Venter WDF, Moorhouse M, Sokhela S (2019). Dolutegravir plus two different prodrugs of tenofovir to treat HIV. N Engl J Med.

[31] Landovitz RJ, Zangeneh SZ, Chau G (2020). Cabotegravir is not associated with weight gain in human immunodeficiency virus-uninfected individuals in HPTN 077. Clin Infect Dis.

[32] Eshleman S, Fogel JM, Halvas EK, et al. CAB-LA PrEP: early detection of HIV infection may reduce InSTI resistance risk. 29th Conference on Retroviruses and Opportunistic Infections; Feb 12–16, 2022 (abstr Oral-08).

