
JOURNAL INFORMATION
==============================
NLM Title Abbreviation: Lancet
Journal ID: Lancet
NLM Journal ID: 2985213R

Lancet (London, England)

ISSN: 0140-6736
EISSN: 1474-547X

ARTICLE INFORMATION
==============================
PMCID: PMC9287682
PMID: 35526551
DOI: 10.1016/S0140-6736(22)00783-8
Article ID: S0140-6736(22)00783-8
Article version: 1
Subjects: Department of Error

Department of Error

Print publication date: 2022 May 7
Volume: 399
Issue: 10337
First page: 1778
Last page: 1778
Copyright: © 2022 The Author(s). Published by Elsevier Ltd.
Copyright year: 2022
Copyright holder: Elsevier Ltd
License: This is an open access article under the CC BY license (http://creativecommons.org/licenses/by/4.0/).
License URL: https://creativecommons.org/licenses/by/4.0/
Erratum for: Cabotegravir for the prevention of HIV-1 in women: results from HPTN 084, a phase 3, randomised clinical trial 399 10337 7 5 2022 1779 1789 Lancet (London, England) 10.1016/S0140-6736(22)00538-4 PMC9077443 35378077

==============================
Delany-Moretlwe S, Hughes JP, Bock P, et al. Cabotegravir for the prevention of HIV-1 in women: results from HPTN 084, a phase 3, randomised clinical trial. Lancet 2022; 399: 1779–89—In this Article, the absolute risk difference between the cabotegravir and TDF-FTC groups in the Findings section of the Summary and the fifth paragraph in the Results should have been –1·6% (–1·0% to –2·3%). This correction has been made to the online version as of May 5, 2022, and the printed version is correct.

