# Supplementary material for: Cabotegravir for the prevention of HIV-1 in women: results from HPTN 084, a phase 3, randomised clinical trial
Source: Lancet. 2022 May 7;399(10337):1779–89. doi: 10.1016/S0140-6736(22)00538-4 (PMC9077443; doi:10.1016/S0140-6736(22)00538-4)
Supplement: Supplementary appendix [file mmc1.pdf]

THE LANCET

Supplementary appendix

This appendix formed part of the original submission and has been peer reviewed. We post it as supplied by the authors.

Supplement to: Delany-Moretlwe S, Hughes J P, Bock P, et al. Cabotegravir for the prevention of HIV-1 in women: results from HPTN 084, a phase 3, randomised clinical trial. *Lancet* 2022; published online April 1. https://doi.org/10.1016/S0140-6736(22)00538-4.

Supplementary figure legends

S1. TFV-DP concentrations by adherence category in a random subset of participants in the TDF/FTC group (n=405 participants)

The figure shows the proportion of randomly selected TDF/FTC subset participants (n=405) with TFV-DP concentrations measured in Dried Blood Spots (DBS) in each adherence category; these data represent average dosing in the previous 1-2 months. Participants could contribute up to 6 samples in this summary; a total of 1197 samples were available for analysis. The number of samples per visit is included in brackets next to the visit week number. For the adherence categories: BLQ <31.25 fmol/punch; <2 doses/week = 31.25-<350 fmol/punch; 2-4 doses/week = 350-<700 fmol/punch; 4-7 doses/week = 700-<1250 fmol/punch; 7 doses/week = >1250 fmol/punch

Abbreviations: BLQ: below the limit of quantitation; TDF/FTC: tenofovir disoproxil fumarate/emtricitabine; TFV-DP: tenofovir diphosphate.

S2. Loess curve of weight gain, by study group

This figure is a smoothed curve fitted through points in a scatter plot that summarise changes in weight (kg) for each participant over time, by study group.

Abbreviations: AEs: adverse events; CAB: cabotegravir, TDF/FTC: tenofovir disoproxil fumarate/emtricitabine

S3. Adverse events according to system organ class categories, by study group.

Severity of adverse events is graded in accordance with the Division of AIDS Table for Grading the Severity of Adult and Paediatric Adverse Events (version 2.1, July 2017). This table includes only those AEs assigned MedDRA codes; injection sites reactions are not included. For participants reporting multiple events of the same MedDRA term, the highest grade is counted. Percentages are calculated as the numbers of participants (n) reporting an AE of specific maximum severity grade divided by the number enrolled by arm.

Abbreviations: CAB: cabotegravir, TDF/FTC: tenofovir disoproxil fumarate/emtricitabine

Figure S1.

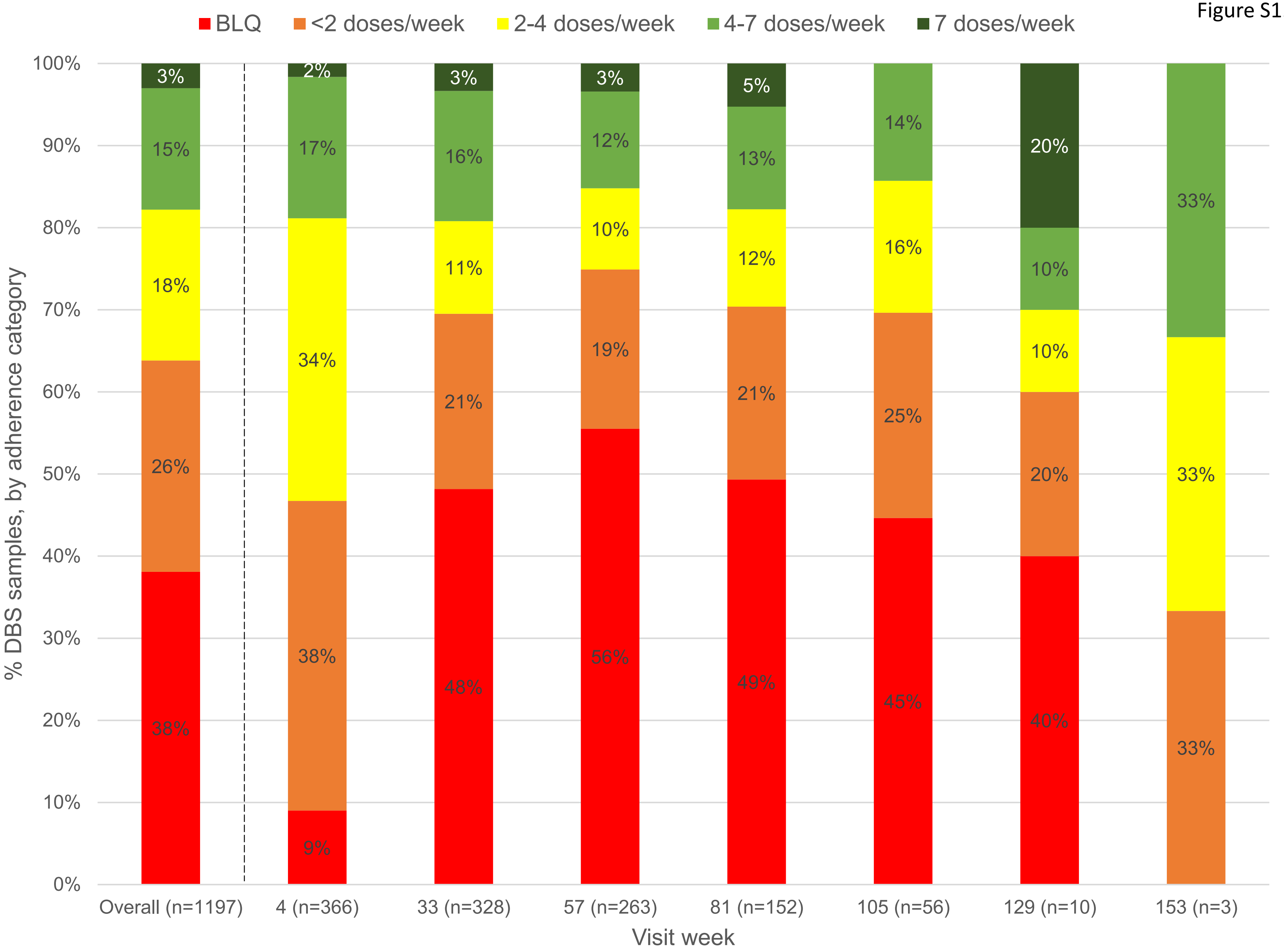

BLQ
<2 doses/week
2-4 doses/week
4-7 doses/week
7 doses/week
100%
90%
80%
70%
60%
50%
40%
30%
20%
10%
0%
% DBS samples, by adherence category
Overall (n=1197)
4 (n=366)
33 (n=328)
57 (n=263)
81 (n=152)
105 (n=56)
129 (n=10)
153 (n=3)
Visit week
3%
15%
18%
26%
38%
2%
17%
34%
38%
9%
3%
16%
11%
21%
48%
3%
12%
10%
19%
56%
5%
13%
12%
21%
49%
14%
16%
25%
45%
20%
10%
10%
20%
40%
33%
33%
33%

Figure. S2

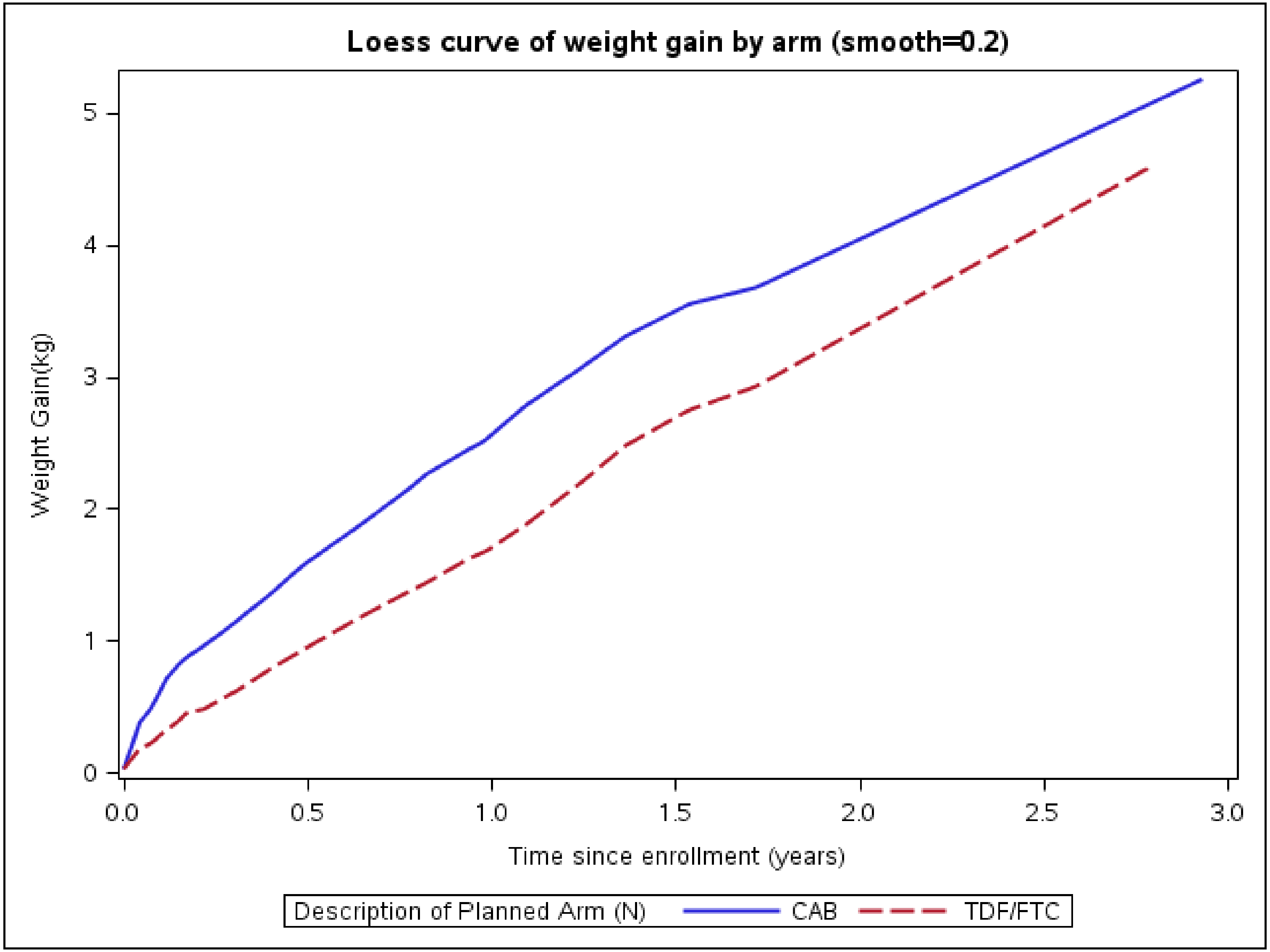

Loess curve of weight gain by arm (smooth=0.2)
Weight Gain(kg)
5
4
3
2
1
0
0.0
0.5
1.0
1.5
2.0
2.5
3.0
Time since enrollment (years)
Description of Planned Arm (N)
CAB
TDF/FTC

Supplementary table 1. Adverse events, by system organ class category

	TDF/FTC						CAB					
	**Overall**	**Grade 1**	**Grade 2**	**Grade 3**	**Grade 4**	**Grade 5**	**Overall**	**Grade 1**	**Grade 2**	**Grade 3**	**Grade 4**	**Grade 5**
**Number of Participants with AEs**	1548 (96.2%)	62 (3.9%)	1206 (74.9%)	241 (15.0%)	39 (2.42%)	0	1559 (96.6%)	72 (4.5%)	1211 (75.0%)	237 (14.68%)	36 (2.2%)	3 (0.2%)
**Blood and lymphatic system disorders**	71 (4.4%)	43 (2.7%)	20 (1.2%)	7 (0.4%)	1 (0.1%)	0	83 (5.1%)	43 (2.7%)	33 (2.0%)	7 (0.4%)	0	0
**Cardiac disorders**	27 (1.7%)	23 (1.4%)	4 (0.3%)	0	0	0	22 (1.4%)	20 (1.2%)	1 (0.1%)	0	0	1 (0.1%)
**Ear and labyrinth disorders**	6 (0.4%)	1 (0.1%)	5 (0.3%)	0	0	0	11 (0.7%)	2 (0.1%)	9 (0.6%)	0	0	0
**Endocrine disorders**	1 (0.1%)	0	1 (0.7%)	0	0	0	1 (0.1%)	0	1 (0.1%)	0	0	0
**Eye disorders**	49 (3.0%)	11 (0.7%)	38 (2.4%)	0	0	0	62 (3.8%)	15 (0.9%)	47 (2.9%)	0	0	0
**Gastrointestinal disorders**	579 (36.0%)	209 (13.0%)	367 (22.8%)	3 (0.2%)	0	0	494 (30.6%)	160 (9.9%)	333 (20.6%)	1 (0.1%)	0	0
**General disorders and administration site conditions**	174 (10.8%)	80 (5.0%)	93 (5.8%)	1 (0.1%)	0	0	160 (9.9%)	85 (5.3%)	75 (4.7%)	0	0	0
**Hepatobiliary disorders**	5 (0.3%)	1 (0.1%)	2 (0.1%)	1 (0.1%)	1 (0.1%)	0	5 (0.3%)	2 (0.1%)	2 (0.1%)	0	1 (0.1%)	0
**Immune system disorders**	42 (2.6%)	5 (0.3%)	36 (2.2%)	1 (0.1%)	0	0	40 (2.5%)	6 (0.4%)	34 (2.1%)	0	0	0
**Infections and infestations**	1041 (64.7%)	54 (3.4%)	962 (59.8%)	23 (1.4%)	2 (0.1%)	0	1049 (65%)	64 (4.0%)	970 (60.1%)	13 (0.8%)	2 (0.1%)	0
**Injury, poisoning and procedural complications**	173 (10.8%)	47 (2.9%)	121 (7.5%)	3 (0.2%)	2 (0.1%)	0	154 (9.5%)	44 (2.7%)	107 (6.6%)	3 (0.20)	0	0
**Laboratory investigations**	1497 (93.0%)	165 (10.3%)	1122 (69.7%)	182 (11.3%)	28 (1.7%)	0	1512 (93.7%)	205 (12.7%)	1085 (67.2%)	191 (11.8%)	31 (1.9%)	0
**Metabolism and nutrition disorders**	353 (21.9%)	159 (9.9%)	150 (9.3%)	42 (2.6%)	2 (0.1%)	0	326 (20.2%)	172 (10.7%)	129 (8.0%)	24 (1.5%)	1 (0.1%)	0
**Musculoskeletal and connective tissue disorders**	270 (16.8%)	50 (3.1%)	220 (13.7%)	0	0	0	317 (19.6%)	87 (5.4%)	229 (14.2%)	1 (0.1%)	0	0
**Neoplasms benign, malignant and unspecified (incl cysts and polyps)**	11 (0.7%)	3 (0.2%)	8 (0.5%)	0	0	0	13 (0.8%)	6 (0.4%)	6 (0.4%)	1 (0.1%)	0	0
**Nervous system disorders**	523 (32.5%)	212 (13.2%)	310 (19.3%)	1 (0.1%)	0	0	526 (32.6%)	215 (13.3%)	307 (19.0%)	2 (0.1%)	0	2 (0.1%)
**Pregnancy, puerperium and perinatal conditions**	7 (0.4%)	3 (0.2%)	3 (0.2%)	0	1 (0.1%)	0	13 (0.8%)	1 (0.1%)	4 (0.3%)	7 (0.4%)	1 (0.1%)	0
**Product issues**	0	0	0	0	0	0	1 (0.1%)	0	1 (0.1%)	0	0	0
**Psychiatric disorders**	75 (4.7%)	39 (2.4%)	30 (1.9%)	2 (0.1%)	4 (0.3%)	0	85 (5.3%)	50 (3.1%)	29 (1.8%)	5 (0.3%)	1 (0.1%)	0
**Renal and urinary disorders**	67 (4.2%)	47 (2.9%)	17 (1.1%)	3 (0.2%)	0	0	66 (4.1%)	48 (3.0%)	13 (0.8%)	5 (0.3%)	0	0
**Reproductive system and breast disorders**	528 (32.8%)	108 (6.7%)	419 (26.2%)	1 (0.1%)	0	0	541 (33.5%)	106 (6.6%)	434 (26.9%)	1 (0.1%)	0	0
**Respiratory, thoracic and mediastinal disorders**	119 (7.4%)	42 (2.6%)	77 (4.8%)	0	0	0	126 (7.8%)	41 (2.5%)	85 (5.3%)	0	0	0
**Skin and subcutaneous tissue disorders**	231 (14.4%)	74 (4.6%)	157 (9.8%)	0	0	0	254 (15.7%)	67 (4.2%)	187 (11.6%)	0	0	0
**Social circumstances**	3 (0.2%)	0	3 (0.2%)	0	0	0	0	0	0	0	0	0
**Vascular disorders**	51 (3.2%)	29 (1.8%)	21 (1.3%)	1 (0.1%)	0	0	62 (3.8%)	49 (3.0%)	13 (0.8%)	0	0	0
